# Development of a Flow-Free Automated Colorimetric Detection Assay Integrated with Smartphone for Zika NS1

**DOI:** 10.3390/diagnostics10010042

**Published:** 2020-01-14

**Authors:** Md Alamgir Kabir, Hussein Zilouchian, Mazhar Sher, Waseem Asghar

**Affiliations:** 1 Department of Computer & Electrical Engineering and Computer Science, Florida Atlantic University, Boca Raton, FL 33431, USA; mkabir2016@fau.edu (M.A.K.); msher2015@fau.edu (M.S.); 2 Asghar-Lab, Micro and Nanotechnology in Medicine, College of Engineering and Computer Science, Boca Raton, FL 33431, USA; hzilouchi@gmail.com; 3 Department of Biological Sciences (Courtesy Appointment), Florida Atlantic University, Boca Raton, FL 33431, USA

**Keywords:** Zika NS1, point of care, colorimetric, smartphone, microfluidic

## Abstract

The Zika virus (ZIKV) is an emerging flavivirus transmitted to humans by *Aedes* mosquitoes that can potentially cause microcephaly, Guillain–Barré Syndrome, and other birth defects. Effective vaccines for Zika have not yet been developed. There is a necessity to establish an easily deployable, high-throughput, low-cost, and disposable point-of-care (POC) diagnostic platform for ZIKV infections. We report here an automated magnetic actuation platform suitable for a POC microfluidic sandwich enzyme-linked immunosorbent assay (ELISA) using antibody-coated superparamagnetic beads. The smartphone integrated immunoassay is developed for colorimetric detection of ZIKV nonstructural protein 1 (NS1) antigen using disposable chips to accommodate the reactions inside the chip in microliter volumes. An in-house-built magnetic actuator platform automatically moves the magnetic beads through different aqueous phases. The assay requires a total of 9 min to automatically control the post-capture washing, horseradish peroxidase (HRP) conjugated secondary antibody probing, washing again, and, finally, color development. By measuring the saturation intensity of the developed color from the smartphone captured video, the presented assay provides high sensitivity with a detection limit of 62.5 ng/mL in whole plasma. These results advocate a great promise that the platform would be useful for the POC diagnosis of Zika virus infection in patients and can be used in resource-limited settings.

## 1. Introduction

The Zika virus (ZIKV) is a flavivirus that is closely related to other flaviviruses such as Dengue, West Nile, and Japanese encephalitis [1]. The virus is primarily spread through a vector, an infected *Aedes* species mosquito, but it can also be transferred through sexual contact and from a pregnant woman to their offspring [2]. Although there were only 1465 reported cases of the Zika virus in the continental USA, Central America, and Mexico, ZIKV still affects 87 countries as of September 2019, according to the World Health Organization (WHO) [3,4]. The ZIKV outbreak in the Americas occurred in 2016 with a steep decline in outbreaks in the following years, where in 2018 only 31,587 reported/probable cases were seen [4]. However, ZIKV is much more prevalent in some Asian and African countries. Indonesia has shown that 9.1% of its population under the age of five has had a prior ZIKV infection. In Lao People’s Republic, 10% of the adult blood donor population showed a prior ZIKV infection [4]. Some symptoms of the ZIKV include mild fever, rash, conjunctivitis, and joint or muscle pain. Many individuals who are infected with ZIKV show no to mild symptoms, which can lead many patients to believe that they are not infected with the virus [5].

The lack of detection of ZIKV in an area cannot be necessarily equated to low levels of transmission or low levels of prevalence. The means of testing in certain areas, especially in rural and third world countries can be lacking, which can attribute to some of these findings. The nonstructural protein 1 (NS1) is targeted for detection due to its important role as a biomarker in flaviviruses. The protein is secreted by cells that contain the virus [6]. As the primary antigen, antibodies to NS1 can be formed in four to seven days, which can be used for detection. Currently the Centers for Disease Control and Prevention (CDC) and WHO guidelines to detect ZIKV are through the use of the nucleic acid amplification test (NAAT) [7]. RT-PCR is presently the most used NAAT within seven days of the onset of symptoms [8]. The problem NAAT faces, in general, is that it is lab based and cannot be performed at point-of-care (POC) settings, it also requires expensive reagents/equipment and technical staff. A negative NAAT) should also be cautiously considered. The IgM antibodies are usually detectable within four to seven days, so the WHO recommends serology testing seven days after the onset of symptoms. A standard enzyme-linked immunosorbent assay (ELISA) test requires 12 hours of testing and the expense of the reagents adds up over time. Therefore, reliable and inexpensive POC testing is of the utmost importance.

IgG and IgM antibodies are produced in the human body at the later stage of the infection (four to seven days), this makes them inapt for early stage detection. In contrast, NS1 antigen with a similar structure has been considered for highly sensitive, specific early stage detection for different flaviviruses previously [9,10,11,12,13,14]. There are currently numerous POC devices that detect ZIKV by using NS1 as a biomarker. The systematic evolution of ligands by exponential enrichment (SELEX) protocol uses aptamers to replace antibodies [10]. The aptamers used allowed the ZIKV NS1 antibody to bind with them, which could be detected through the use of ELISA. Although the technique lowered the cost and sensitivity, the assay is not suitable for point-of-care applications. Paper-based lateral flow immunoassay (LFIA) devices also exist, which can be effective in detecting infections/molecules [11,15,16,17,18]. Even though LFIA is cheap, rapid, and portable, it can only give qualitative or semiqualitative results. A laser cut glass fiber paper-based analytical device called PAD based on lateral flow technique has shown a limit of detection of around 25 ng/mL and can be used for NS1 biomarker detection in low-resource areas. The PAD assay provides only qualitative results, which can be done in 10 min [19]. However, the PAD requires several manual steps as well as a heating (60 °C) step to perform the assay.

In this paper, we have developed a microfluidic magnetic ELISA (M-ELISA) system that provides benefits over other POC testing and traditional testing through its low cost, time efficiency, and automation. Compared to conventional sandwich ELISA, the unique shape of the microfluidic chip that we have developed can decrease cost due to the use of less reagents and time by cutting the test from 12 hours to approximately 10 min. This can be beneficial in certain parts of the world where accessibility and cost are significant factors. The reduction in time is achieved through the increase of surface area with the magnetic beads [20]. The developed device is also automated, which can create a significant advantage and allow an individual to run multiple assays at a time.

## 2. Experimental Section

### 2.1. Sandwich ELISA for Detecting Zika NS1 Antigen in 96-Well-Plate Format

To validate the Antigen- Antibody (Ag–Ab) reaction, sandwich ELISA assay was first performed by coating a microplate with 100 μL of 2 μg/mL capture antibody for Zika NS1 (BF-1225-36, Biofront Technologies, Tallahassee, Florida, USA) in carbonate/bicarbonate buffer (pH 9.6), and then incubating it overnight at 4 °C. After washing three times with phosphate buffered saline (PBS) (pH 7.4), each well was blocked by 200 μL of SuperBlock T20 (PBS) Blocking Buffer (37517, Fisher Scientific, Hampton, NH, USA) for 90 min on a 15 rpm shaker at room temperature. It was rewashed three times carefully, followed by 90 min incubation of 100 μL of recombinant Zika NS1 antigen (BF-NS1-6309, Biofront Technologies, Tallahassee, Florida, USA) with different concentrations spiked in PBS at room temperature. One hundred microliters of HRP-labeled anti-Zika NS1 antibody (BF-1125-36-HRP, Biofront Technologies, Tallahassee, Florida, USA) with 1:1000 dilution buffer (PBS containing 0.02% Tween 20, 3% Bovine Serum Albumins (BSA)) was added and incubated for 60 min on a 15 rpm shaker at room temperature followed by three washes with PBS. Blue color development was carried out using a 3,3′,5,5′-tetramethylbenzidine (TMB) substrate (PI34028, Fisher Scientific, Hampton, New Hampshire, USA) by incubating for 10 min in a dark place, and 2M H_2_SO_4_ was used to stop the color development. The absorbance was measured at 450 nm using a SpectraMax Gemini™ XPS/EM Microplate Reader (Molecular Devices, San Jose, CA, USA).

### 2.2. Microfluidic Chip Design

We fabricated a three-layer microchip using a cheaper non-lithographic method by laser cutting (Universal Laser) polymethylmethacrylate (PMMA) and double-sided adhesive (DSA) materials using an optimized design previously published [20]. The top layer (750 µm thick) has 0.4 mm diameter inlets and outlets to fit the pipette tips to allow easy sample loading. The middle layer (1.5 mm) contains reservoirs for all the mineral oil and aqueous solution, and the bottom layer (750 µm thick), which is solid, works as a base for the microchip. All the layers are assembled by using DSA films (Supplementary Figure S2a,b), and then pressed uniformly to remove all the bubbles by a bench vise (Home Depot).

### 2.3. Conjugation of Magnetic Beads with Capture Antibody

The capture antibody was first biotinylated using a type B fast biotin conjugation kit (ab201796) obtained from Abcam. One hundred microliters of Zika NS1 Mab (1225-36, Biofront Technologies) was modified by 10 µL of biotin-modified reagent and agitated very gently. The antibody mixer was added to the lyophilized biotin vial (100 µg), followed by 15 min incubation at room temperature. Ten microliters of Quencher reagent was added to stop the biotin reaction and was incubated for 4 min. Magnetic particles with an average diameter of 1 μm coated with neutravidin (GE Healthcare, Chicago, IL, USA) show higher affinity surface functionalization for biotinylated antibodies. Four hundred microliters of neutravidin-coated magnetic beads with 3500–4500 picomole/mg binding capacity were washed with 4 mL of PBS in a 5 mL Eppendorf Protein LoBind tube (14-282-304, Fisher Scientific). A magnetic stand was used for attracting all the magnetic beads creating a pellet on the magnetic stand side tube wall. The supernatant was discarded using a pipette and substituted by the same amount of PBS and mixed gently with a pipette to wash the magnetic beads. The process was repeated two times. For Zika NS1 capturing, 4 mL of 25 μg/mL biotinylated Zika NS1 Mab (1225-36, Biofront Technologies) was conjugated with the neutravidin-coated magnetic beads. To create a magnetic bead–biotin–capture antibody conjugation, the mixer was incubated overnight on a shaker (15 rpm) at 4 °C. After incubation; the magnetic bead conjugation was washed three times following the earlier mentioned steps to remove boundless Zika NS1 antibodies. One hour of blocking was done by using SuperBlock T20 (PBS) Blocking Buffer (37517, Fisher Scientific) to functionalize the magnetic beads at room temperature followed by washing twice using 4 mL PBS. After rinsing the magnetic beads, the conjugation was resuspended in 4 mL PBS and stored at 4 °C for future use.

### 2.4. M-ELISA on 96-Well Plate

Before using the magnetic bead–antibody solution, the beads were vortexed for 2–3 s to make a homogeneous solution. Thirty microliters of (1 mg/mL) magnetic beads conjugated with capture antibody was added on a conical-bottom 96-well plate (12-565-215, Fisher Scientific), and then all supernatant was isolated and discarded with the help of a 96-well-plate magnetic separator stand. One hundred microliters of Zika NS1 antigen spiked in plasma was added on to the wells and mixed gently to create a homogeneous solution followed by a 45 min incubation at room temperature on a shaker at 15 rpm speed. After that, the beads were aggregated by applying them onto the magnetic separator for 30–40 s to allow the magnetic beads to create a pellet on the sidewall of the wells. The liquid was discarded by a pipette and replaced with 200 µL of PBS to wash the beads. Beads were washed three times carefully to remove all the uncaptured antigen. One hundred microlites of HRP-labeled anti-Zika NS1 antibody (BF-1125-36-HRP, Biofront Technologies Tallahassee, Florida, USA) with 1:1000 dilution buffer (PBS containing 0.02% Tween 20, 3% BSA) was added and mixed slowly and incubated for 15 min on a 15 rpm shaker at room temperature. The beads were again washed three times by following the procedure mentioned above. One hundred microliters of TMB substrate was added, mixed, and incubated for 90 s in a dark place to generate a blue color. Color development was stopped by using 100 μL of 2M H_2_SO_4_. Beads were isolated with the help of a magnetic separator, and the liquid was transferred to a new well and absorbance was measured at 450 nm by using a SpectraMax Gemini™ XPS/EM Microplate Reader (Molecular Devices, San Jose, CA, USA).

### 2.5. Automated M-ELISA on Chip

**Figure 1:**
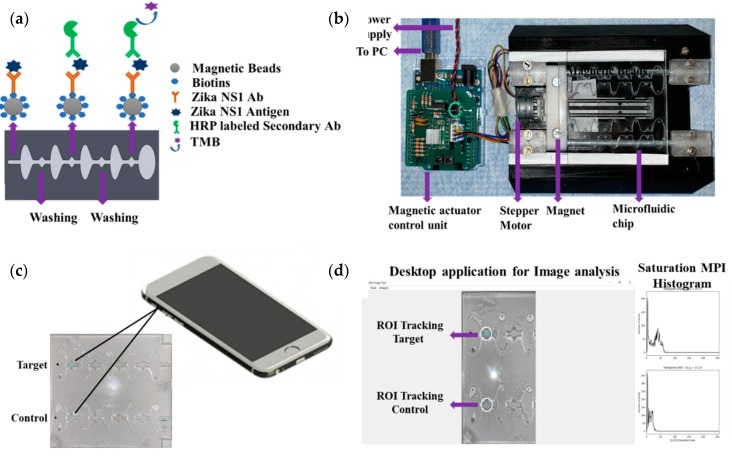
Graphical representation of the microfluidic sandwich enzyme-linked immunosorbent assay (ELISA) inside of a microfluidic chip. (**a**) Captured antigen is loaded inside the chip and moved through a washing buffer. The antigen-captured beads are labeled with the HRP-conjugated secondary antibody in the next chamber. After moving through a washing buffer chamber again, blue color was developed by reacting with a color-generation substrate. The reaction was stopped by moving the beads to a retention chamber. (**b**) A magnetic actuation platform containing an Arduino-controlled stepper motor unit. The 3D printed platform facilitates stepper motor housing allowing the user to move the magnets according to the command set up using a PC. The platform also holds the microfluidic chip just above the magnets. (**c**) Video frames of developed color on the chip are directly captured by a smartphone. (**d**) Region-of-interest (ROI) tracking using a desktop application and a histogram plot of the saturation maximum pixel intensity (MPI) of the developed color by the microfluidic magnetic enzyme linked immunosorbent assay (M-ELISA) on chip.

An Arduino-controlled in-house-built magnetic actuation platform [20] was used to facilitate the automation of the developed assay (Figure 1a,b). The platform consisted of two parts including an Arduino controlling unit and the 3D printed platform, which could accommodate the microfluidic chip. The control unit housed the stepper motor driver (Pololu A4988) to regulate the bidirectional motor movement through a linear slide enclosed with two magnets (Supplementary Movie S1). The controlled unit was commanded by a gcode script set using a computer interface and powered by an external power supply.

The prefabricated three-layer microfluidic chip was first loaded with all the reagents. First, 40 µL of (1 mg/mL) magnetic beads conjugated with capture antibody were taken into a 0.5 mL protein LoBind PCR tube, and then the supernatant was isolated with the help of a magnet and discarded by a pipette. One hundred microliters of Zika NS1 antigen diluted 2000-62.5 ng/mL concentration was added to the PCR tube and mixed gently. Then the PCR tube was incubated for 10 minutes at room temperature on a 15 rpm shaker to allow the beads to capture the antigen. In the meantime, the other reagents were loaded into the microfluidic chip with the help of a pipette. The loading steps in brief were as follows (Supplementary Figure S1), first both of the washing chambers (1,2) were filled with PBS, then HRP-labeled anti-Zika NS1 antibody (1:500 diluted in PBS containing 0.02% Tween 20, and 3% BSA) was loaded in chamber 3 on the chip followed by TMB substrate loading on the color-generation chamber, chamber 4. The TMB substrate was loaded in a dark place, and, after loading, the chamber was covered by an opaque tape to avoid oxidation with the reaction of normal light. After that, mineral oil (Sigma Aldrich, St. Louis, Missouri, USA) with a viscosity of 15 cst was loaded in chambers 5, 6, 7 and 8. After the Zika NS1 capture in the PCR tube, magnetic beads were isolated, and the supernatant was removed. Thirty microliters of PBS was replaced on the tube and mixed gently with the beads. Then the beads solution was loaded into chamber 9. Finally, chip loading was completed by loading mineral oil in chamber 10. The full loading process took 6–7 min. After loading the chip, it was placed on the magnetic actuation platform to perform washing, labeling with HRP-conjugated anti-Zika NS1 antibody, and color development, automatically (Figure 1a). The M-ELISA on-chip steps, in brief, were as follows, the magnets placed under the chip, which was enclosed by stepper motor, first moved inside chamber 9 for 15 s to accumulate all the beads and create a pellet. Then the beads were washed in chamber 1 for one minute to remove any uncaptured NS1 proteins. After washing, the beads were moved to chamber 3, where the beads were probed with HRP-labeled anti-Zika NS1 antibody. To remove all the nonspecifically bounded HRP-labeled anti-Zika NS1, the beads were washed again for one minute in chamber 2. The blue color was generated in the chamber 4 by reacting with TMB substrate for 30 s. The fully automated ELISA process took approximately 19–20 min including the sample preparation and loading.

### 2.6. Image Acquisition and Analysis

The microfluidic chip was placed on a white paper on a flat surface instantly after the completion of the M-ELISA. The covering tape was removed as quickly as possible, and a 30 s video was recorded using iPhone Xs cellphone (Figure 1c). The camera flashlight was on during the video recording (30 fps) to avoid external light interference and light variation from external sources. The white paper under the chip provided uniform background conditions. An OpenCV- and Python-based image processing semiautomated windows desktop application was used for image analysis (Figure 1d). First, the video was segregated frame by frame using a MATLAB (MathWorks, Natick, MA, USA) code and each 30th frame (equal to 1 second) was considered to select 30 images from the total 30 s video. Each image was analyzed by the desktop application to quantify the saturation maximum pixel intensity (MPI) in sequence. To analyze the intensity of color change, we manually selected the region of interest (ROI) using the desktop application. The desktop application facilitates the user to draw, drag, and drop a circle on top of the ROI of the analyzed images (Figure 1d). The arithmetic mean of the saturation channel was calculated during the colorimetric analysis. In this analysis, the ROI was chosen based on the portion having maximum saturation intensity for both sample and control channels. To set the sensitivity of the assay, the mean saturation pixel value of negative control (Dengue 2 NS1) as a result of M-ELISA on a 96-well plate was considered as the basis (mean ±3 standard deviation).

## 3. Results and Discussion

**Figure 2:**
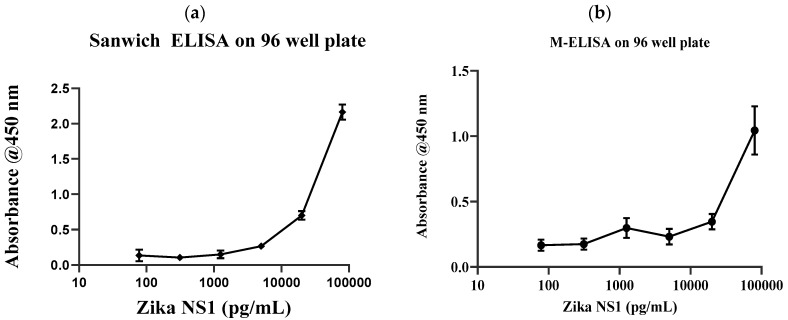
(**a**) Sandwich ELISA assay results using the recombinant Zika nonstructural protein 1 (NS1) antigen spiked on PBS buffer on 96-well plate as the Zika NS1 monoclonal antibody is used as a capture agent. Anti-Zika NS1 monoclonal antibody-HRP with a dilution factor of 1:1000 utilized to react with TMB calorigenic substrate to develop color. (**b**) M-ELISA assay on a 96-well plate showing spiking recombinant Zika NS1 on whole plasma. A 1:500 dilution factor of HRP-labeled anti-Zika NS1 was used and reacted with TMB to generate color. In both cases, color development was stopped by using H_2_SO_4_ and absorbance was measured at 450 nm using a SpectraMax Gemini™ XPS/EM Microplate Reader (Molecular Devices, USA). Error bars are ±SD.

Conventional gold standard sandwich ELISA was carried out on a 96-well plate with multiple dilutions of Zika NS1 antigens (78.5 pg/mL–80 ng/mL) to validate the antibody and the target binding. Results clearly show that the anti-Zika NS1 monoclonal antibody was binding as low as 78 pg/mL of Zika NS1 when it was spiked in PBS (Figure 2a) with a higher correlation (R^2^ ≥ 0.9929). In this study, we used Dengue 2 NS1 antigen as negative control and we also performed standard sandwich ELISA to further confirm that there was no cross reactivity/false-positive result shown by the anti-Zika NS1 monoclonal antibody (Supplementary Figure S3).

**Figure 3:**
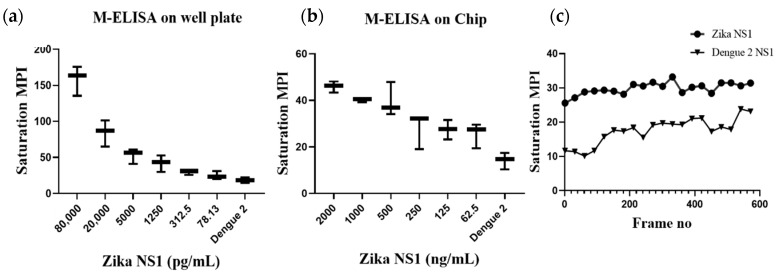
Standard curve of saturation MPI (**a**) for a captured cell phone image of the 96-well plate after performing M-ELISA on the 96-well plate and (**b**) M-ELISA on chip by computing the developed color intensity of the segmented frames of a captured video. An OpenCV- and Python-based semiautomated desktop application were used to select the ROI to calculate the saturation MPI. The use of the cell phone quantitation method on an M-ELISA showing a limit of detection of 78 pg/mL (R^2^ ≥ 0.9561) and 62.5 ng/mL (R^2^ ≥ 0.9929) on 96-well plate and microfluidic chip, respectively. Error bars are ±SD. (**c**) Comparison of saturation MPI change over time, quantified by analyzing a 20 s video (30 fps) just after completing the M-ELISA on chip. The full video was segmented frame by frame, and each 30th frame was analyzed to compare the saturation MPI change over frames (time).

After validating the Ag–Ab reaction, the anti-Zika NS1 monoclonal antibody was conjugated with superparamagnetic beads using a commercially available biotin conjugation kit. To confirm the magnetic bead–biotin–antibody binding, we performed a zeta potential analysis. A change in the negative surface charge indicates that the beads were successfully conjugated with antibodies. Supplementary Figure S4 shows that the surface charge of the magnetic beads was −5.63 mV and it was changed to −21.4 mV after the beads–antibody conjugation. With the antibody-conjugated beads, we developed and optimized a protocol for the M-ELISA to be performed on a 96-well plate. Again, different concentrations of Zika NS1 protein (78.5 pg/mL–80 ng/mL) spiked in whole plasma were used for the standard curve readouts; whereas Dengue 2 NS1 antigen spiked in plasma was used as a negative control. We also optimized the dilution factor of HRP-labeled anti-Zika NS1 antibody for the M-ELISA (not reported) to reduce the background readout. The absorbance reading was taken by the SpectraMax Gemini™ XPS/EM Microplate Reader. A picture of the 96-well plate was taken with camera flashlight on. After extracting the saturation MPI of the taken image, it confirmed that both the M-ELISA standard curve (Figure 2b), as well as the desktop application, generated the saturation MPI standard curve (Figure 3a) and presented comparable results. The observed detection limit of Zika NS1 in M-ELISA on 96-well plate was 78 pg/mL (R^2^ ≥ 0.9929). This result indicates that the M-ELISA is also consistent like conventional sandwich ELISA. Therefore, the measurement of the saturation MPI to calculate the concentration of Zika NS1 is also reliable.

To further reduce the required reagent and assay timing for performing the M-ELISA we used a previously reported [20] Arduino-controlled magnetic actuation platform (Figure 1b).

To fabricate the three layers of microfluidic chip to accommodate different reagents, we chose 0.75 mm thick PMMA for the top and bottom layers and a 1.5 mm middle layer (Supplementary Figure S2). The total volume of the chip was 907 µL, and it contained a total of 10 chambers. Our designed chip has been optimized such that (Supplementary Figure S2c) it will have the highest bead-to-magnet attraction, low bead loss while moving from one chamber to another chamber, and reliable quantification based on previously published work [20,21]. Three differently shaped chambers were used while designing the microfluidic chips. The cylindrical chamber is considered as the bead aggregation chamber and the magnetic actuation starts from this chamber. In this chamber, the magnet oscillates two times for approximately 16 seconds to facilitate creation of the bead pellet. The four diamond-shaped chambers contain washing buffer, HRP-labeled anti NS1 antibody, and TMB substrates. All the elliptical chambers contained mineral oil which basically acts as a physical barrier between each aqueous reagent. The shapes are designed and optimized in a way that provides minimum surface tension and minimum meniscus effect while the magnetic bead moves from one phase to another linearly.

After validating target Ag–Ab binding by conventional sandwich ELISA and M-ELISA (Figure 2a,b), the application of the M-ELISA on the chip was performed using the automated platform. The assay run time was optimized to 9 min and 20 min (Table 1) including sample preparation and loading. Until now the Zika NS1 levels in Zika-infected patients were still largely unknown/variable, whereas approximately 15 μg/mL Dengue NS1 can be found for Dengue-infected patient’s serum after two days of infection [12,22]. Based on that, we have analyzed 2000.0, 1000.0, 500.0, 250.0, 125.0, and 62.5 ng/mL Zika NS1 spiking on plasma to perform M-ELISA on-chip (Figure 3b). The observed detection limit for the M-ELISA on-chip was 62.5 ng/mL.

To the best of our evidence, this is the first reported automated sandwich ELISA technique for detecting Zika NS1 antigen. Our goal was to develop and confirm the sandwich ELISA with superparamagnetic beads on a microfluidic chip. The non-lithographic process of chip fabrication has been reported previously [21,23,24,25] for inexpensive, disposable, and robust applications for various analyte detections in resource-limited settings. Supplementary Table S1 shows the manufacturing cost for each developed microfluidic chip and the cost per assay. We used smartphone captured video and an in-house-developed desktop application to calculate the color development by TMB. Video capture instead of a single-picture capture provides the flexibility of choosing a large set of images taken over a period of time [26]. In this study, video capture plays an important role as there was no stopping solution after the TMB reaction. After the completion of beads’ movement and color development, the color was changing until saturation. Since saturation can measure the intensity of the color, it is a suitable measure for concentration-dependent colorimetric assays, and it has been reported previously [26,27]. Saturation represents the amount of intensity of color in an image and can be represented from 0 to 255 in binary scale. The intensity of color development is correlated with the concentration-dependent colorimetric assays. While considering the image for calculating the saturation MPI for both samples and control, each 30th frame was considered to record the color development. Unlike conventional sandwich ELISA, our M-ELISA chip method contains no stopping solution. As a result of this, the developed color intensity increases over the video capturing time. Figure 3c shows the relation between both samples and control color change over time. While quantifying the saturation MPI, we consider the mean ±3 standard deviation of the control’s saturation MPI of M-ELISA on a 96-well plate as a basis to determine the sensitivity irrespective of any specific frame or time of the captured video.

Recently, very few studies have been published for developing detection assays targeting ZIKV NS1 antigen. A double antibody sandwich ELISA-based colorimetric assay was able to detect it as low as 120 ng/mL [6]. Another sensing platform using a graphene biosensor was able to detect 500 ng/mL spiked in a 10-fold diluted serum [28]. An antigen–antibody-based, rapid paper-based diagnostic assay has been reported to have a sensitivity of about 20 ng/mL for ZIKV NS1 [29]. Following an alternative approach, our developed assay was able to detect as low as 62.5 ng/mL in whole plasma, which is comparable to other currently existing or developed techniques.

Moreover, the developed assay is suitable for detecting the Zika NS1 antigen without the help of costly specialized instruments (e.g., a microplate reader), which in turn reduces cost per assay to below $2 (USD) (Supplementary Table S1). The unique features for the developed assays are automation, rapid assay turnout, and result readout by cell-phone-based video capture. Sample handling and loading do not require any skilled personnel. The developed device design is highly scalable and microfluidic chips can be designed to run up to 12 assays in one go by further optimization. To further reduce assay loading time, preloaded devices can be designed and tested. Considering the throughput against 96-/384-well plate-based conventional sandwich ELISA, our developed platform has lower throughput but considering other factors especially portability, cost, and applicability it is suitable for developing countries where resources are limited, and a smaller number of tests are required per day. The developed assay fulfills ASSURED criteria [30] (i.e., affordable, sensitive, specific, user-friendly, rapid, equipment-free, and deliverable) for being applicable to the POC.

## 4. Conclusions

We developed an automated microfluidic assay integrated with smartphone that can obtain results in ~10 minutes, which can be beneficial in resource-limited settings. There were some shortcomings and further areas for our device to expand on. The sensitivity of our device is much higher than that of other point-of-care devices with a detection limit of 62.5 ng/mL. Further improvement in this area can result in great potential for this device to be clinically used in areas around the world. However, the cost effectiveness, automation, and time effectiveness compared to traditional ELISA’s can be beneficial in regions that struggle with poverty and lack of resources. The utilization of smartphone video and the in-house software can be operated by low-skilled workers. This, in turn, can further the accessibility of the developed device and allow anyone to record and interpret results. Without the use of high-cost specialized instruments, the developed chip can be kept at a low cost. These results show potential that the platform would be useful for the POC diagnosis/screening of Zika virus infection patients and can be used in resource-limited settings.

## Figures and Tables

**Table 1:** Time comparison of sandwich ELISA, M-ELISA, and M-ELISA on chip.

Steps		Sandwich ELISA	M-ELISA	M-ELISA on Chip
*Time consumption*	Coating	Overnight	Pre-prepared	Pre-prepared
	Antigen capture	1.5 h	45 min	10 min (outside chip)
	Blocking	1.5 h	–	–
	Secondary antibody labeling	1 h	15 min	5 min
	Development	10 min	1.5 min	30 s
	Washing	20 min	10 min	2 min
	Beads collection and moving	–	–	1 min
	Total Time	~4.5 h	~72 min	~9 min
*Sample consumption (*µL*)*	Coating	100	Pre-prepared	Pre-prepared
	Antigen Capture	100	100	~30
	Blocking	200	Pre-prepared	Pre-prepared
	Secondary antibody labeling	100	100	~60
	Development	100	100	~60
	H_2_SO_4_	100	100	–
	Washing	1500	900	~120
	Separation (oil)	–	–	~464
	Retention (oil)	–	–	~173
	Total reagent	2200	1300	~907
Limit of detection		78 pg/mL	78 pg/mL	62.5 ng/mL

## Supplementary Materials

The following are available online at https://www.mdpi.com/2075-4418/10/1/42/s1, Table S1: Material and reagents cost of the disposable microfluidic chip, Figure S1: Reagent Loading sequence inside the microfluidic chip, Figure S2: Chip design, fabrication, and assembly, Figure S3: Sandwich ELISA assay for Dengue 2 NS1 with the capture antibody for Zika NS1 for detection in binding buffer, Figure S4: Zeta potential measurement of antibody coated beads, Video S1: Sample reagent loading and test run.

## References

[1] Sikka V., Chattu V.K., Popli R.K., Galwankar S.C., Kelkar D., Sawicki S.G., Stawicki S.P., Papadimos T.J. (2016). The Emergence of Zika Virus as a Global Health Security Threat: A Review and a Consensus Statement of the INDUSEM Joint Working Group (JWG). J. Glob. Infect. Dis..

[2] Herrada C.A., Kabir M., Altamirano R., Asghar W. (2018). Advances in Diagnostic Methods for Zika Virus Infection. J. Med. Devices.

[3] PAHO, WHO Cases of Zika Virus Disease. http://www.paho.org/data/index.php/en/?option=com_content&view=article&id=524&Itemid=.

[4] WHO Zika Epidemiology Update. https://www.who.int/emergencies/diseases/zika/zika-epidemiology-update-july-2019.pdf?ua=1.

[5] Musso D., Roche C., Robin E., Nhan T., Teissier A., Cao-Lormeau V.-M. (2015). Potential sexual transmission of Zika virus. Emerg. Infect. Dis..

[6] Zhang L., Du X., Chen C., Chen Z., Zhang L., Han Q., Xia X., Song Y., Zhang J. (2018). Development and characterization of double-antibody sandwich ELISA for detection of zika virus infection. Viruses.

[7] World Health Organization (2016). Laboratory Testing for Zika Virus Infection: Interim Guidance.

[8] Landry M.L., George K.S. (2016). Laboratory diagnosis of Zika virus infection. Arch. Pathol. Lab. Med.

[9] Matheus S., Boukhari R., Labeau B., Ernault V., Bremand L., Kazanji M., Rousset D. (2016). Specificity of dengue NS1 antigen in differential diagnosis of dengue and Zika virus infection. Emerg. Infect. Dis..

[10] Lee K.H., Zeng H. (2017). Aptamer-based ELISA assay for highly specific and sensitive detection of Zika NS1 protein. Anal. Chem..

[11] Rong Z., Wang Q., Sun N., Jia X., Wang K., Xiao R., Wang S. (2019). Smartphone-based fluorescent lateral flow immunoassay platform for highly sensitive point-of-care detection of Zika virus nonstructural protein 1. Anal. Chim. Acta.

[12] Sánchez-Purrà M., Carré-Camps M., de Puig H., Bosch I., Gehrke L., Hamad-Schifferli K. (2017). Surface-enhanced raman spectroscopy-based sandwich immunoassays for multiplexed detection of zika and dengue viral biomarkers. ACS Infect. Dis..

[13] Wang S.M., Sekaran S.D. (2010). Evaluation of a commercial SD dengue virus NS1 antigen capture enzyme-linked immunosorbent assay kit for early diagnosis of dengue virus infection. J. Clin. Microbiol..

[14] Cecchetto J., Fernandes F.C., Lopes R., Bueno P.R. (2017). The capacitive sensing of NS1 Flavivirus biomarker. Biosens. Bioelectron..

[15] Chen Y., Wang Y., Liu L., Wu X., Xu L., Kuang H., Li A., Xu C. (2015). A gold immunochromatographic assay for the rapid and simultaneous detection of fifteen β-lactams. Nanoscale.

[16] Song S., Liu N., Zhao Z., Njumbe Ediage E., Wu S., Sun C., De Saeger S., Wu A. (2014). Multiplex lateral flow immunoassay for mycotoxin determination. Anal. Chem..

[17] Zhang L., Huang Y., Wang J., Rong Y., Lai W., Zhang J., Chen T. (2015). Hierarchical Flowerlike Gold Nanoparticles Labeled Immunochromatography Test Strip for Highly Sensitive Detection of Escherichia coli O157:H7. Langmuir.

[18] Choi J.R., Yong K.W., Tang R., Gong Y., Wen T., Yang H., Li A., Chia Y.C., Pingguan-Murphy B., Xu F. (2017). Lateral Flow Assay Based on Paper–Hydrogel Hybrid Material for Sensitive Point-of-Care Detection of Dengue Virus. Adv. Healthc. Mater..

[19] Theillet G., Rubens A., Foucault F., Dalbon P., Rozand C., Leparc-Goffart I., Bedin F. (2018). Laser-cut paper-based device for the detection of dengue non-structural NS1 protein and specific IgM in human samples. Arch. Virol..

[20] Coarsey C., Coleman B., Kabir M.A., Sher M., Asghar W. (2019). Development of a flow-free magnetic actuation platform for an automated microfluidic ELISA. RSC Adv..

[21] Wang S., Tasoglu S., Chen P.Z., Chen M., Akbas R., Wach S., Ozdemir C.I., Gurkan U.A., Giguel F.F., Kuritzkes D.R. (2014). Micro-a-fluidics ELISA for Rapid CD4 Cell Count at the Point-of-Care. Sci. Rep. Artic..

[22] Steinhagen K., Probst C., Radzimski C., Schmidt-Chanasit J., Emmerich P., van Esbroeck M., Schinkel J., Grobusch M.P., Goorhuis A., Warnecke J.M. (2016). Serodiagnosis of Zika virus (ZIKV) infections by a novel NS1-based ELISA devoid of cross-reactivity with dengue virus antibodies: A multicohort study of assay performance, 2015 to 2016. Eurosurveillance.

[23] Asghar W., Sher M., Khan N.S., Vyas J.M., Demirci U. (2019). Microfluidic Chip for Detection of Fungal Infections. ACS Omega.

[24] Sher M., Asghar W. (2019). Development of a multiplex fully automated assay for rapid quantification of CD4+ T cells from whole blood. Biosens. Bioelectron..

[25] Wang S., Zhao X., Khimji I., Akbas R., Qiu W., Edwards D., Cramer D.W., Ye B., Demirci U. (2011). Integration of cell phone imaging with microchip ELISA to detect ovarian cancer HE4 biomarker in urine at the point-of-care. Lab-on-a-Chip.

[26] Coleman B., Coarsey C., Kabir M.A., Asghar W. (2019). Point-of-care colorimetric analysis through smartphone video. Sens. Actuators B Chem..

[27] Coleman B., Coarsey C., Asghar W. (2019). Cell phone based colorimetric analysis for point-of-care settings. Analyst.

[28] Afsahi S., Lerner M.B., Goldstein J.M., Lee J., Tang X., Bagarozzi D.A., Pan D., Locascio L., Walker A., Barron F. (2018). Novel graphene-based biosensor for early detection of Zika virus infection. Biosens. Bioelectron..

[29] Bosch I., De Puig H., Hiley M., Carré-Camps M., Perdomo-Celis F., Narváez C.F., Salgado D.M., Senthoor D., O’Grady M., Phillips E. (2017). Rapid antigen tests for dengue virus serotypes and Zika virus in patient serum. Sci. Transl. Med..

[30] Peeling R.W., Mabey D., Herring A., Hook E.W. (2006). Why do we need quality-assured diagnostic tests for sexually transmitted infections?. Nat. Rev. Microbiol..

