# Supplementary material for: Development of a Flow-Free Automated Colorimetric Detection Assay Integrated with Smartphone for Zika NS1
Source: Diagnostics (Basel). 2020 Jan 14;10(1):42. doi: 10.3390/diagnostics10010042 (PMC7168132; doi:10.3390/diagnostics10010042)
Supplement: Supplementary file 1 [file diagnostics-10-00042-s001.pdf]

Supplementary information

Development of an inexpensive flow-free automated lab-on-chip colorimetric detection assay for Zika NS1

Md Alamgir Kabir,[1,2] Hussein Zilouchian,[2] Mazhar Sher,[1,2] Waseem Asghar[1,2,3,*]

[1]Department of Computer & Electrical Engineering and Computer Science, Florida Atlantic University, Boca Raton, FL 33431

[2]Asghar-Lab, Micro and Nanotechnology in Medicine, College of Engineering and Computer Science, Boca Raton, FL 33431

[3]Department of Biological Sciences (Courtesy Appointment), Florida Atlantic University, Boca Raton, FL 33431

* Corresponding Author Email: wasghar@fau.edu

Supplementary Table 1: Material and reagents cost of the disposable microfluidic chip.

Items	*Cost/Chip*
Poly(methyl methacrylate) (PMMA)	***$ 0.15***
Double Sided Adhesive ((DSA)	***$ 0.05***
Chip reagents	***$ 0.95***
Magnetic Beads	***$0.40***
Mineral oil	***$ 0.05***
Total	***$ 1.60***

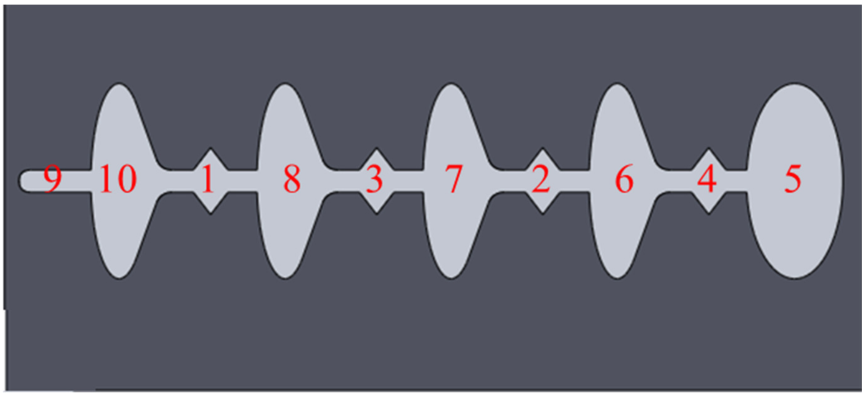

Supplementary Figure 1: Reagent Loading sequence inside the microfluidic chip. The schematic shows the chronological order of loading reagents into the microfluidic chip. 1, 2- PBS (washing buffer), 3- HRP labeled secondary antibody, 4- TMB substrate, 5,6,7,8 – mineral oil, 9- Captured antigen conjugated with anti-Zika antibody, and 10- mineral oil. Each chamber (not shown) has 2 holes with 0.4 mm diameter which acts as sample loading and pressure releasing holes.

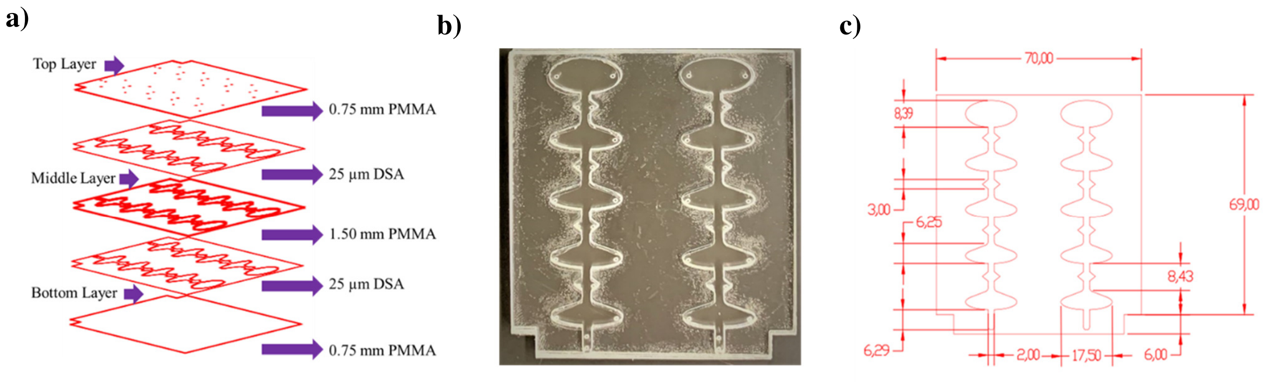

Supplementary Figure 2: Chip design, fabrication, and assembly. a) Assembly of three layer microfluidic chips with PMMA and DSA. The PMMA and DSA schematics are designed in AutoCAD and cut by CO2 laser cutter. DSA layers works as cement and holds other PMMA layers. b) Microfluidic chip after full assembly layer by layer. c) The optimized design of the chambers and their dimensions in mm.

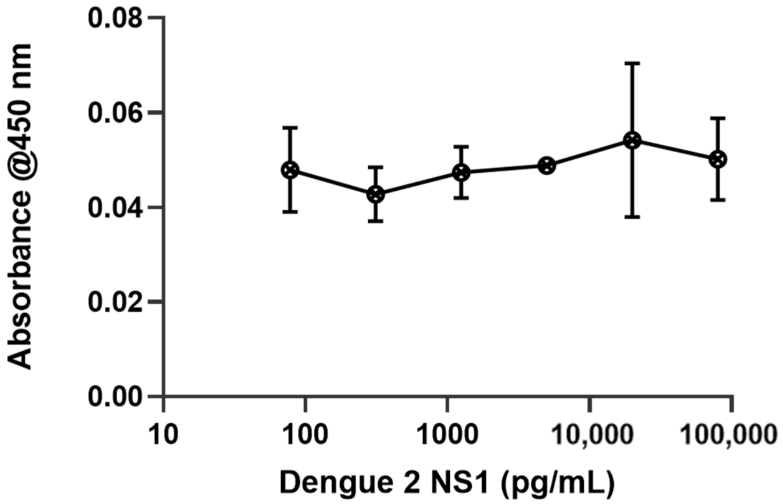

Supplementary Figure 3: Sandwich ELISA assay for Dengue 2 NS1 with the capture antibody for Zika NS1 for detection in binding buffer. Confirmation of non-cross reactivity of Dengue 2 NS1 antigen spiked in buffer with Anti Zika NS1 monoclonal antibody by standard Sandwich ELISA. HRP labeled Anti-Zika NS1 was used for the color development. After color development using TMB-based substrate and stop solution, absorbance was measured at 450nm using SpectraMax Gemini™ XPS/EM Microplate reader (Molecular Devices, USA). X axis is in log10 scale. Error bars are ±SD

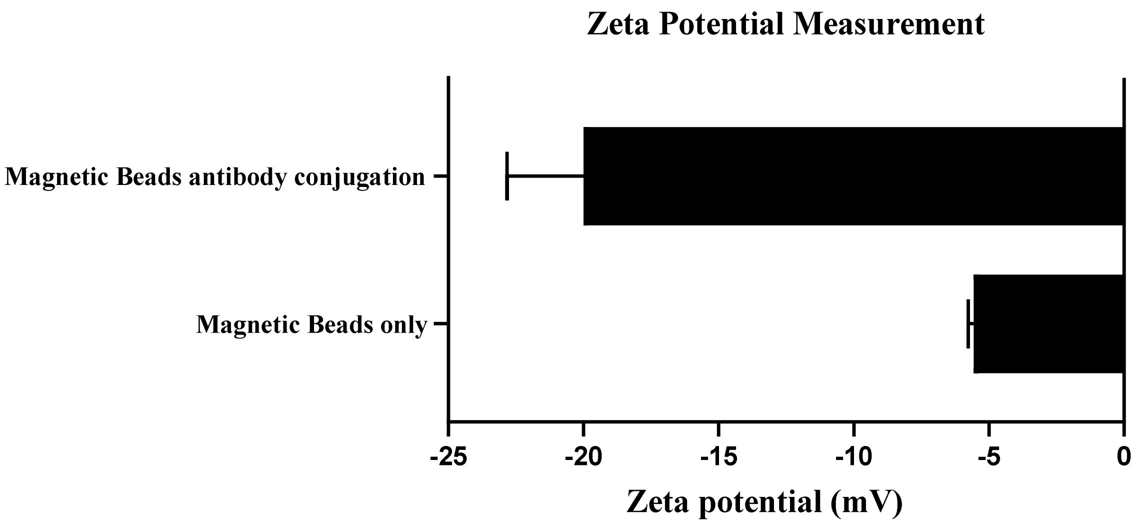

Supplementary Figure 4: Zeta potential measurement of antibody coated beads. Zeta Potential values of magnetic beads before and after conjugation with antibody. The zeta potential of the beads was measured by using Malvern Zetasizer Nano ZS device.
